# PD-1 Inhibitors Plus Capecitabine as Maintenance Therapy for Advanced Intrahepatic Cholangiocarcinoma: A Case Report and Review of Literature

**DOI:** 10.3389/fimmu.2021.799822

**Published:** 2021-12-24

**Authors:** Zhihong Wang, Tianmei Zeng, Yong Li, Ding Zhang, Zhengang Yuan, Mengli Huang, Yuan Yang, Weiping Zhou

**Affiliations:** ^1^ Eastern Hepatobiliary Surgery Hospital, Second Military Medical University, Shanghai, China; ^2^ The Medical Department, 3D Medicines Inc., Shanghai, China

**Keywords:** intrahepatic cholangiocarcinoma (iCCA), immunotherapy, maintenance therapy, biomarker (BM), capecitabine

## Abstract

Intrahepatic cholangiocarcinoma (iCCA) is the second most common primary liver cancer with a poor prognosis. Recently, an immunotherapy strategy represented by programmed cell death 1 (PD-1) inhibitors has been applied to the systemic treatment of advanced iCCA. However, immunotherapy combined with chemotherapy as first-line maintenance therapy was rarely reported. Our report presented an advanced iCCA patient who had a dramatic response to the PD-1 inhibitor sintilimab combined with gemcitabine plus cisplatin as the first-line therapy and sintilimab combined with capecitabine as maintenance therapy, yielding an ongoing progression-free survival of 16 months.

## Introduction

Intrahepatic cholangiocarcinoma (iCCA) is an aggressive malignancy that originates in the bile duct epithelium within the liver. Gemcitabine plus cisplatin (GC regimen) as a first-line standard of care offers a limited median overall survival (OS) of approximately 12 months for advanced iCCA patients (1). Immune checkpoint inhibitors (ICIs), such as programmed cell death 1 (PD-1) inhibitors and programmed cell death ligand 1 (PD-L1) inhibitors, have demonstrated substantial benefits in multiple disease settings with significant efficacy and manageable toxicity (2). Compared to chemotherapy, the combination of ICIs and GC regimen as the most common therapeutic strategy significantly improved the survival of patients with advanced iCCA. However, capecitabine in combination with ICIs as a first-line maintenance therapy without progression was rarely reported for advanced iCCA. Here we presented a case of advanced iCCA that responded dramatically to the PD-1 inhibitor sintilimab combined with GC regimen as first-line systemic therapy. After eight cycles of treatment, the combination of sintilimab and capecitabine was administered as maintenance therapy. The tumor lesions disappeared completely, and the patient had a long-term benefit. In our case, PD-1 inhibitor-involved maintenance therapy in patients with advanced iCCA was reported for the first time, and the combination provided a therapeutic strategy for clinical consideration.

## Results

### Case Presentation

A 60-year-old man was found to have liver-occupying lesions by color Doppler ultrasound. The patient has no viral hepatitis, biliary stones, non-alcoholic fatty liver disease, or family history of disease. Computed tomography (CT) revealed a lesion in the caudate lobe of the right lobe of the liver by 83 mm × 72 mm. Heterogeneous enhancement and enlarged lymph nodes were observed. Here, 2-[^18^F]-fluoro-2-deoxy-d-glucose (18F-FDG) uptake increased, and the standardized uptake value (SUV) was 18.6. The metabolic activity of 18F-FDG in the axillary, mediastinum, armpit, hilum, retroperitoneum, and pleura also increased, and the maximum SUV was 13.3, considering tumor metastasis. Carcinoembryonic antigen (CEA) and alpha-fetoprotein (AFP) levels were within the normal range, while cancer antigen 19-9 (CA19-9) level was increased to 2,168 U/ml. The immunohistochemical (IHC) analysis revealed the following: CK7 (+), CK19 (+), Muc-1 (+), Hep-1 (-), Arginase (-), Gly-3 (-), GS (-), CK5/6 (+), and p40 (-). These results suggested a diagnosis of stage IV iCCA (pT4N1M1).

**Figure 1:**
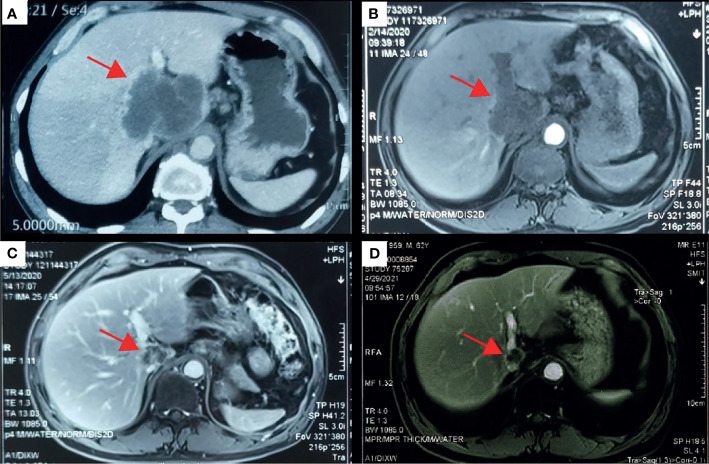
Computed tomography (CT) scans revealed the changes of the primary lesion over time. **(A)** CT results (December 5, 2019) before first-line therapy (sintilimab combined with gemcitabine plus cisplatin). **(B)** CT results (February 14, 2020) at the first imaging evaluation. **(C)** CT results (May 13, 2020) at the end of the first-line systemic therapy. **(D)** CT results (April 19, 2021) at the latest imaging evaluation of the maintenance therapy.

**Figure 2:**
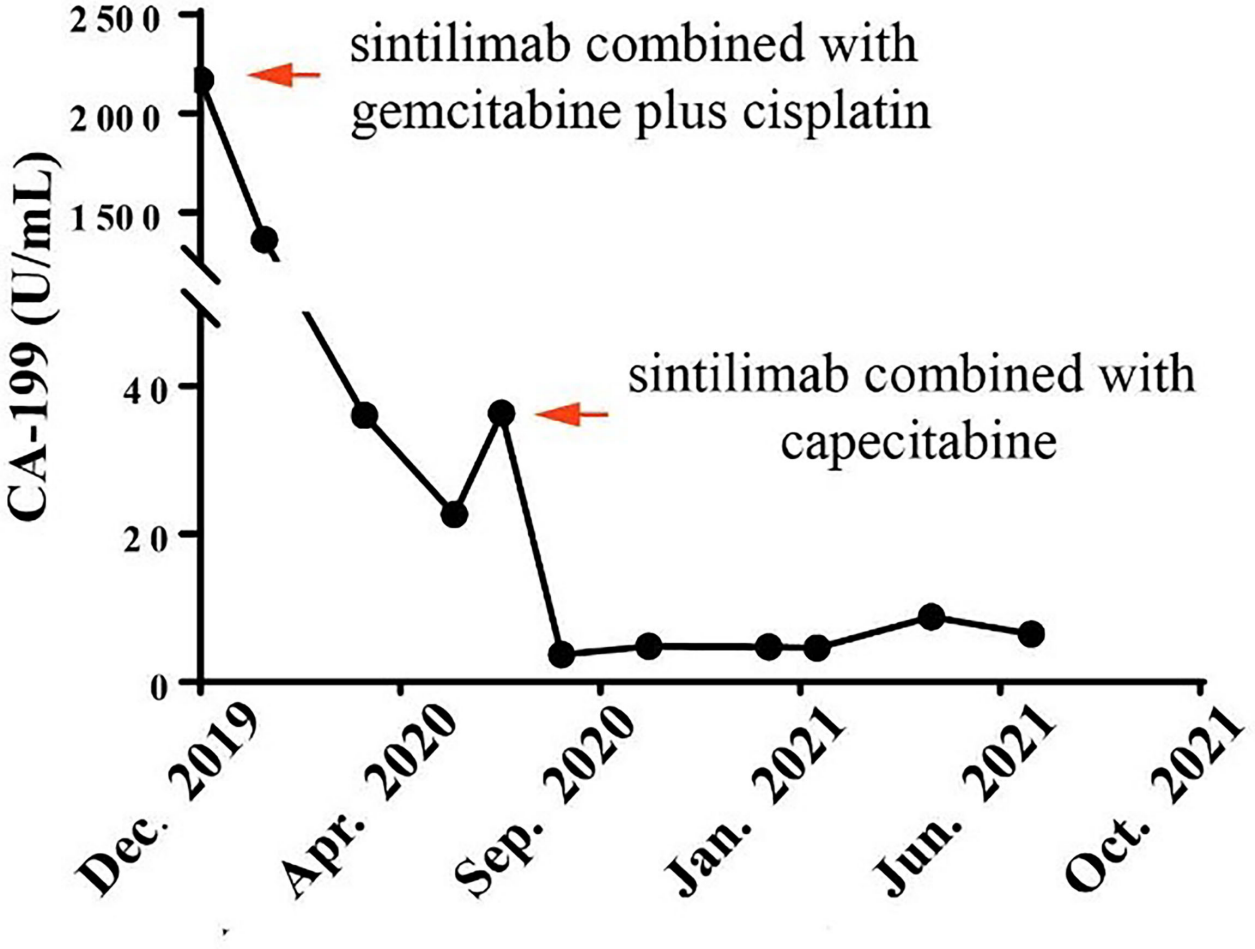
Course of cancer antigen 19-9 (CA19-9) levels in relation to applied therapies: course of tumor markers over time. Arrows indicate application of first-line therapy (sintilimab combined with gemcitabine plus cisplatin) and maintenance therapy (sintilimab combined with capecitabine).

With the patient’s consent, the tissue sample obtained during surgery was subjected to next-generation sequencing (NGS) using a 733-gene panel and IHC detection of PD-L1 performed in a College of American Pathologists (CAP)- and Clinical Laboratory Improvement Amendments (CLIA)-certificated lab. Somatic gene mutations have been detected in tissue, including *APC* with an allelic fraction of 8.06% and *ARID1A* with an allelic fraction of 5.76%. In addition, the patient had a *BRCA2* p.R2659G germline mutation. NGS results indicated the low-level microsatellite instability and the intermediate tumor mutational burden (TMB; 7.26 Mutants/Mb) (**Table 1**). IHC results showed that the PD-L1 tumor proportion score was less than 1%, which indicated low expression of PD-L1. The patient had a strong will to receive immunotherapy or targeted therapy. However, no approved targeted drug was available for the gene mutations detected by NGS. *ARID1A* mutation was correlated with high immune infiltrates in most of the cancer types. Several studies suggested the improved effect of ICIs in tumors with *ARID1A* mutation (3, 4). With this patient’s informed consent and requirement, we considered sintilimab combined with the GC regimen as the first-line treatment strategy. The patient was started on sintilimab (200 mg one cycle for 21 days) combined with cisplatin (40 mg/m^2^ one cycle for 21 days) plus gemcitabine (1,600 mg/m^2^ one cycle for 21 days) in December 2019. Within the first 2 months of treatment, CT results showed that the tumor size was decreased from 83 mm × 72 mm to 64 mm × 64 mm (**Figures 1A, B** **)**. Then the tumor had shrunk gradually. In May 2020, CT results showed that the huge tumor had shrunk to 22.5% of the size before treatment (**Figure 1C**). No systemic abnormalities occurred, and the CA19-9 level dropped within the normal range. After eight cycles, the CA 19-9 level increased slightly. Considering the convenience and comfort of administration, the patient stopped GC regimen injection and took capecitabine 1,250 mg/m^2^ orally. Sintilimab was kept on as the previous dose. He tolerated the medication without any adverse effects. The CA19-9 level stayed below 10 U/mL (**Figure 2**). The patient experienced an elevation of alanine aminotransferase (ALT) in December 2019 and dropped from 117 to 16 U/L in May 2020. Aspartate aminotransferase (AST) was abnormal (111 U/L) in July 2021, followed by normal 2 months later. ALT and AST at other times were in the normal range during the follow-up. Other liver function indicators, such as total bilirubin, direct bilirubin, and albumin, were in normal range during the treatment. To date, there is no further enlargement of the primary tumor (**Figure 1D**). The metastases had disappeared entirely. The progression-free survival (PFS) has already exceeded 16 months.

**Table 1:** Summary of gene test results and mutations that may have clinical significance.

Summary of gene test results							
Somatic mutations that may have clinical significance	Germline mutations that may have clinical significance	Tumor mutational burden	PD-L1 Immunohistochemical results (Dako 22C3 antibody)	Microsatellite analysis	Tumor cell content	Specimen type
2 mutations: APC, ARID1A	1 mutation: BRCA2	7.26 Muts/Mb Intermediate (lower than 31% iCCA)	Negative (TPS <1%, CPS = 1)	MSS	60%	FFPE tissue
**Somatic mutations that may have clinical significance**						
**Gene**		**Exon**		**Protein**		**Mutation abundance**
ARID1A		Exon 1		p.S262*		5.76%
APC		Exon 16		p.E2015Sfs*23		8.06%
**Germline mutations that may have clinical significance**						
**Gene**		**Exon**		**Protein**		**Mutation abundance**
BRCA2		Exon 17		p.R2659G		50%

iCCA, intrahepatic cholangiocarcinoma; MSS, microsatellite-stable; FFPE, formalin-fixed and paraffin-embedded; PD-L1, programmed cell death-Ligand 1; CPS, Combined Positive Score; TPS, Tumor cell Proportion Score.

* is used for nucleotide numbering and indicates the termination codons translation.

## Discussion

Advanced iCCA is clinically aggressive in digestive tract tumors, with the median survival of approximately 1 year. GC regimen remains the primary treatment in advanced iCCA. So far, no PD-1 inhibitor has been approved for the first-line systemic treatment of this disease except pembrolizumab for mismatch repair-deficient (dMMR) or microsatellite instability (MSI) high (MSI-H) tumors. Although not yet recommended by the National Comprehensive Cancer Network (NCCN) guidelines, several clinical studies and case reports of a PD-1 inhibitor in combination with chemotherapy used for advanced biliary tract cancers have been reported (5–8). However, the clinical benefit of PD-1 inhibitor-involved maintenance therapy without progression in patients with advanced iCCA is uncertain. Little is known about the clinical effect of PD-1 inhibitors combined with capecitabine in advanced iCCA. We reported a case of advanced iCCA that responded to sintilimab combined with GC regimen as first-line systemic therapy, followed by sintilimab plus capecitabine as maintenance therapy. The PFS has been more than 16 months.

The immunotherapy strategy recommended by NCCN guidelines of biliary tract cancers (version 3. 2021) included pembrolizumab for tumors with dMMR/MSI-H or high TMB (TMB-H), lenvatinib combined with pembrolizumab, and nivolumab monotherapy for subsequent-line therapy. Recently, the clinical studies of PD-1 inhibitors combined with chemotherapy brought promising results. In the subsequent-line therapy of biliary tract cancers, the median OS with a PD-1 inhibitor plus chemotherapy was 14.9 months, 8 months longer than those with PD-1 inhibitor monotherapy and chemotherapy alone (6). A phase II trial of biliary tract cancers demonstrated that patients who received nivolumab plus GC regimen as first-line treatment had an objective response rate (ORR) of 61.9%, a median PFS of 6.2 months, and a median OS of 8.6 months (5). The PD-1 inhibitor plus chemotherapy might improve the clinical response rate for patients with positive PD-L1 expression or TMB-H (7, 9). However, our report showed that the PD-L1 expression of the patient was negative, and the TMB level was intermediate. According to the review of literature, *ARID1A* mutation was associated with the outcomes of ICIs. In a retrospective study involving 112 patients with hepatobiliary malignancies, patients with *ARID1A* mutation had longer PFS than those without *ARID1A* mutation [hazard ratio (HR) = 0.62; 95% CI 0.40–0.97; *p* = 0.03] after immunotherapy. Notably, none of the patients with hepatobiliary tumors was MSI-H or TMB-H (3). The predictive value of *ARID1A* mutation on immunotherapy was also confirmed in other tumor types (3, 4). The potential mechanism was considered to be that patients with *ARID1A* mutation had markedly high immune infiltrates, especially CD8+ T cells (4, 10). In this case, the patient had an *ARID1A* mutation and reached a PFS of 16 months, which suggested that *ARID1A* mutation might be a potential biomarker for ICIs in iCCA, regardless of MSI, PD-L1, or TMB status.

There is no standard maintenance treatment for advanced iCCA. A retrospective study of biliary tract cancers demonstrated that the median PFS with GC maintenance regimen was 2.8 months longer than that, with observation in patients showing no progression after first-line GC regimen (11). Maintenance therapy with gemcitabine alone was well tolerated and resulted in a significant clinical delayed response in a case with metastatic iCCA (12). The PACIFIC study reported that durvalumab following concurrent chemotherapy with thoracic radiation (CRT) was well tolerated, with a long-term survival benefit (13). Thus, the PACIFIC regimen was established as the standard of care in patients with unresectable stage III non–small-cell lung cancer who did not progress while undergoing CRT. For the first time, we tried PD-1 inhibitor combined with chemotherapy as first-line maintenance therapy in advanced iCCA.

Capecitabine is a medicine for oral administration used for iCCA therapy. Capecitabine plus cisplatin had the same effectiveness as GC regimen in patients with advanced biliary tract cancers (14). However, grade 3/4 toxicities were significantly less frequent in patients treated with capecitabine plus cisplatin than patients treated with the GC regimen (24.9% vs. 40.9%, *p* = 0.002) (15). Considering the convenience and comfort of administration, we used capecitabine plus sintilimab as maintenance therapy. This case innovatively reported on PD-1 inhibitor combined with capecitabine as a treatment strategy in advanced iCCA.

## Conclusion

In conclusion, this is the first case describing the clinical benefit of PD-1 inhibitor-involved maintenance therapy without progression in patients with advanced iCCA, demonstrating a potential treatment strategy of the PD-1 inhibitor plus capecitabine. *ARID1A* mutation may be a key factor in the superior outcome of immunotherapy. However, it is only a case report, and the results need to be further explored with larger sample sizes.

## Methods

The patient received the NGS of a tissue sample with 733 cancer-related gene panel in a CAP- and CLIA-certificated lab to seek potential treatment opportunities. The somatic and germline mutation data were both obtained. Only the pathogenic mutations and likely pathogenic mutations in clinical significance were rolled into our analysis. PD-L1 expression status was determined by immunohistochemistry using the Dako 22C3 assay.

## Data Availability Statement

The raw data supporting the conclusions of this article will be made available by the authors without undue reservation.

## Ethics Statement

Ethical review and approval were not required for the study on human participants in accordance with the local legislation and institutional requirements. The patients/participants provided their written informed consent to participate in this study. Written informed consent was obtained from the individual for the publication of any potentially identifiable images or data included in this article.

## Author Contributions

YY, ZW, and YL contributed to the data collection. YY and TZ contributed to the paper assessment. DZ, ZY, and MH contributed to article writing. YY and WZ contributed to article revising. All authors had read and approved the final version. All authors contributed to the article and approved the submitted version.

## Funding

This work was supported by the Science Fund for Creative Research Groups, NSFC, China (81521091), and Clinical Research Plan of SHDC (SHDC22020213).

## Conflict of Interest

Authors DZ and MH are employed by 3D Medicines Inc.

The remaining authors declare that the research was conducted in the absence of any commercial or financial relationships that could be construed as a potential conflict of interest.

## Publisher’s Note

All claims expressed in this article are solely those of the authors and do not necessarily represent those of their affiliated organizations, or those of the publisher, the editors and the reviewers. Any product that may be evaluated in this article, or claim that may be made by its manufacturer, is not guaranteed or endorsed by the publisher.
